# Influence of citrate concentration on the activation of blood cells in an *in vitro* dialysis setup

**DOI:** 10.1371/journal.pone.0199204

**Published:** 2018-06-13

**Authors:** Jakob Gubensek, Karin Strobl, Stephan Harm, Rene Weiss, Tanja Eichhorn, Jadranka Buturovic-Ponikvar, Viktoria Weber, Jens Hartmann

**Affiliations:** 1 Department of Nephrology, University Medical Center Ljubljana, Ljubljana, Slovenia; 2 Faculty of Medicine, University of Ljubljana, Ljubljana, Slovenia; 3 Center for Biomedical Technology, Department for Health Sciences and Biomedicine, Danube University Krems, Krems, Austria; 4 Christian Doppler Laboratory for Innovative Therapy Approaches in Sepsis, Danube University Krems, Krems, Austria; University of Rochester Medical Center, UNITED STATES

## Abstract

**Background:**

Regional citrate anticoagulation has been associated with enhanced biocompatibility in hemodialysis, but the optimal dose of citrate remains to be established. Here, we compared parameters related to cellular activation during *in vitro* dialysis, using two doses of citrate.

**Methods:**

Human whole blood, anticoagulated with either 3 mM or 4 mM of citrate, was recirculated in an *in vitro* miniaturized dialysis setup. Complement (C3a-desArg), soluble platelet factor 4 (PF4), thromboxane B2 (TXB2), myeloperoxidase (MPO), as well as platelet- and red blood cell-derived extracellular vesicles (EV) were quantified during recirculation. Dialyzer fibers were examined by scanning electron microscopy after recirculation to assess the activation of clotting and the deposition of blood cells.

**Results:**

Increases in markers of platelet and leukocyte activation, PF4, TXB2, and MPO were comparable between both citrate groups. Complement activation tended to be lower at higher citrate concentration, but the difference between the two citrate groups did not reach significance. A strong increase in EVs, particularly platelet-derived EVs, was observed during *in vitro* dialysis for both citrate groups, which was significantly less pronounced in the high citrate group at the end of the experiment. Assessment of dialyzer clotting scores after analysis of individual fibers by scanning electron microscopy revealed significantly lower scores in the high citrate group.

**Conclusions:**

Our data indicate that an increase in the citrate concentration from 3 mM to 4 mM further dampens cellular activation, thereby improving biocompatibility. A concentration of 4 mM citrate might therefore be optimal for use in clinical practice.

## Introduction

Regional citrate anticoagulation (RCA) has found wide-spread application in centrifugal apheresis and has become the preferred method of anticoagulation for continuous renal replacement therapy (CRRT) due to its safety and effectiveness [1]. RCA has been proposed to provide optimal anticoagulation for chronic hemodialysis patients with increased bleeding risk [2] and represents one of the two methods recommended by the current guidelines in this situation [3].

By chelating calcium and magnesium, RCA minimizes the risk of clotting in the extracorporeal circuit inherent to heparin-free dialysis and enables safe maintenance hemodialysis. Citrate is thought to have benefits beyond anticoagulation [4] and has been reported to reduce complement activation [5] as well as platelet and leukocyte activation [6–9], thereby dampening inflammatory reactions in the extracorporeal circuit and ensuring an extended filter lifetime during continuous treatment [10, 11]. There is evidence that many of the inhibitory effects of citrate in the extracorporeal circuit are dose-dependent [12], and clinical data suggest that higher doses of citrate are required to improve blood compatibility [6, 7, 13]. A number of protocols for RCA have been proposed which differ with respect to the citrate dose in addition to other technical details, yet the optimal dosing of citrate in clinical practice remains to be established. According to published protocols, citrate doses in the extracorporeal circuit range from 2.4–2.7 mM [13–15] to 3–3.5 mM [4, 6, 7]. Doses above 4 mM [16–18] up to 6.7 mM have been applied as well [19], usually in combination with lower blood flows to reduce the amount of citrate delivered to the patient [16, 17, 19].

Citrate from the extracorporeal circuit is mainly removed in the dialyzer, and residual citrate infused into the patient is metabolized to bicarbonate by the liver and by the skeletal muscles with a half-life of 30–60 min. While citrate dosing has to ensure adequate anticoagulation of the extracorporeal circuit, the rate of citrate infused into the patient should be kept low to limit potential systemic effects of re-infused citrate, such as hypocalcemia or metabolic alkalosis, in particular in patients with impaired citrate metabolism. In this context, we assessed the effect of different citrate concentrations on the activation of blood cells in an *in vitro* dialysis setup and evaluated the influence of citrate on cellular adhesion and on the release of potentially thrombogenic extracellular vesicles.

## Materials and methods

### Ethical statement

Blood collection was approved by the Institutional Review Board of Danube University Krems, and written informed consent was obtained from all donors.

### Chemicals and reagents

Unfractionated heparin was purchased from Gilvasan Pharma, Vienna, Austria. Phosphate buffered saline (PBS) was from Thermo Fisher Scientific, Waltham, MA. Glutaraldehyde was obtained from Roth, Karlsruhe, Germany. Ethylenediaminetetraacetic acid (EDTA) dihydrate and Indomethacin were purchased from Sigma Aldrich, Vienna, Austria. Tri-sodium citrate (0.5 M) was purchased from Provobispharm, Bad Ischl, Austria.

### Human blood

Venous blood was freshly drawn from healthy adult volunteers for each experiment and used within less than 30 min for *in vitro* dialysis. Blood was collected into two separate blood bags (Fresenius Medical Care, Bad Homburg, Germany; 2 x 200 ml), connected with a y-piece in the tubing line. Bags were prefilled with trisodium citrate and heparin to achieve a final concentration of either 3 mM or 4 mM of citrate and 3 IU of heparin per liter of blood.

### *In vitro* dialysis

**Fig 1:**
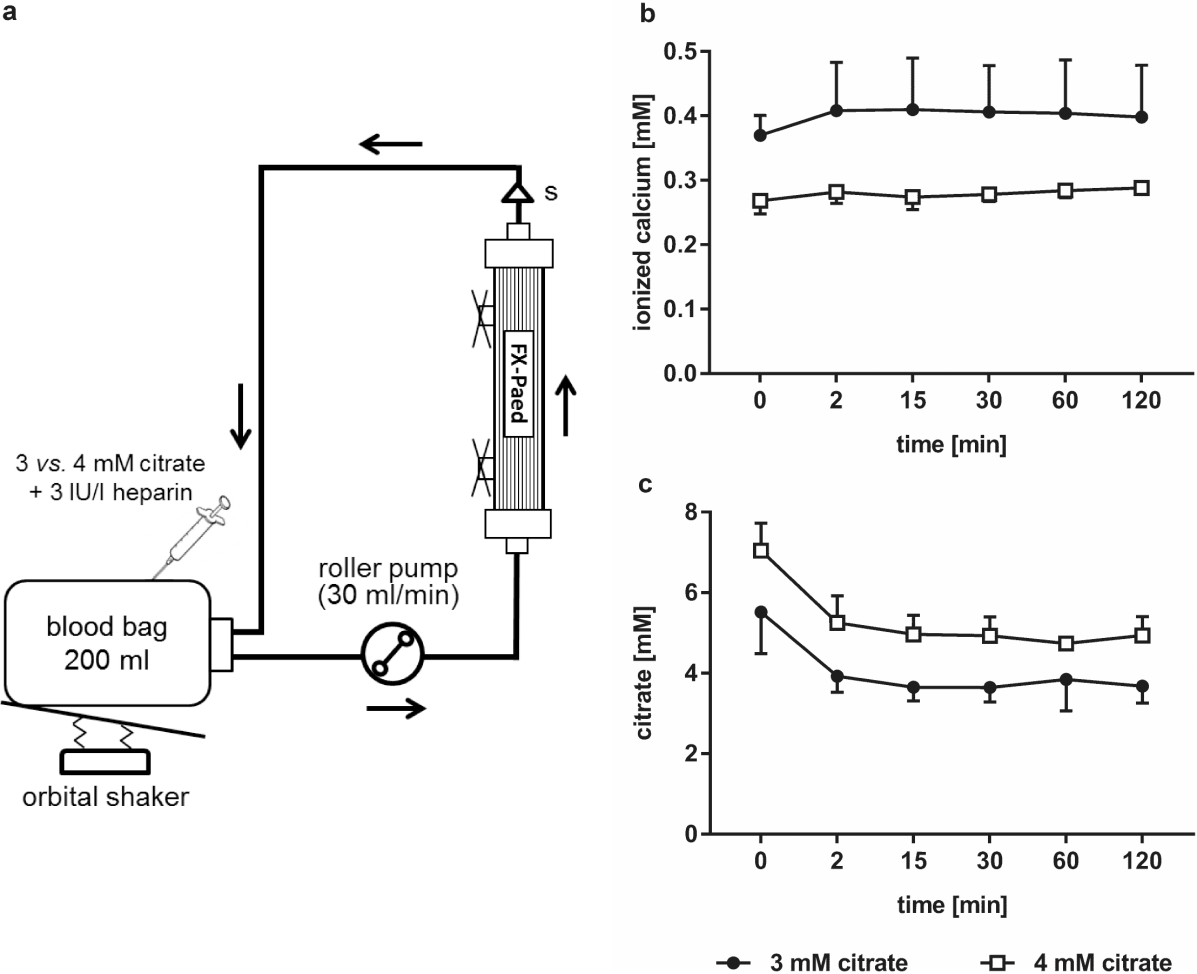
Scheme of the experimental set-up and levels of ionized calcium and citrate over the course of the experiment. (a) Freshly drawn whole blood was anticoagulated with either 3 mM or 4 mM citrate supplemented with 3 IU/l unfractionated heparin and recirculated as described in the Methods section. Samples were taken at the indicated time points after the dialyzer (s) and analyzed for biocompatibility parameters as shown in Fig 2; (b) levels of ionized calcium, and (c) citrate levels over the course of the experiment.

Two identical hemodialysis circuits composed of a pediatric tubing system (AV-Set-FMC Paed/Baby R, Fresenius Medical Care), from which the venous chamber was excluded, a pediatric dialyzer (FX Paed, Fresenius Medical Care), and a roller pump (Ismatec IP65, Wertheim, Germany) were set up in advance and rinsed with isotonic saline solution (Fig 1A). The dialysate connectors on the dialyzers were closed and the experiment was run without dialysate. Baseline blood samples were drawn from the blood bags before the start of the recirculation. Blood was simultaneously introduced via the arterial line of both circuits, and 40 ml of saline, corresponding to the approximate volume of the extracorporeal system, was discarded from the circuits before the venous line was connected to the blood donation bag. Blood was recirculated at 30 ml/min at 37°C, and the blood bags were gently moved with an orbital shaker to avoid sedimentation of the blood cells. Blood samples were drawn after the dialyzer at 2, 15, 30, 60, and 120 min and either analyzed directly or used to generate plasma for further analysis as stated for the individual parameters below. After 120 min, both systems were prepared for electron microscopy as described below. Five independent experiments were performed.

### Basic laboratory parameters

Ionized calcium and magnesium were quantified in whole blood using an ion-selective electrode (NOVA Biomedical, Waltham, MA). Blood cell counts were determined using a Sysmex KX-21N analyzer (Kobe, Japan). For quantification of citrate, human whole blood samples were centrifuged and the resulting plasma was stored at -20°C until analysis. Citrate concentration was determined using an enzymatic method (R-Biopharm, Darmstadt, Germany) on an automated analyzer (Cobas c311, Roche Diagnostics, Rotkreuz, Switzerland).

### Biocompatibility parameters

Complement activation was determined by quantification of C3a-desArg by ELISA (Progen Biotechnik, Heidelberg, Germany). Samples were immediately aliquoted into tubes containing 6.7 mM EDTA to prevent post sampling complement activation, centrifuged at 2,000 g (10 min, 4°C) to obtain plasma, and stored at -80°C until analysis. Platelet activation was assessed by quantification of soluble platelet factor 4 (PF4) and thromboxane B2 (TXB2) by ELISA (R&D Systems, Minneapolis, MN). Samples for TXB2 determination were aliquoted into tubes containing indomethacin to prevent further generation of TXB2. Leukocyte activation was assessed by quantification of plasma myeloperoxidase (MPO) by ELISA (Immun Diagnostik, Bensheim, Germany). For all assays, blood samples were centrifuged at 2000 g (10 min, 4°C), and plasma was stored at -20°C until analysis.

### Characterization of extracellular vesicles

As an additional parameter indicating cellular activation, the release of extracellular vesicles (EVs) during *in vitro* dialysis was assessed. Platelet free plasma was generated from whole blood samples by immediate centrifugation at 2,500 g for 10 min at room temperature and re-centrifugation of the supernatant at 13,000 g for 15 min at room temperature. Plasma samples were stored at -80°C until further analysis, and EVs were characterized using a Gallios Flow Cytometer (Beckman Coulter, Brea, CA). Calibration was performed with fluorescent beads (0.1, 0.3, 0.5, and 0.9 μm; Megamix Plus FSC, Biocytex, Marseille, France) according to the instructions of the manufacturer, and the EV gate was set above the 0.9 μm bead cloud as described [20–22]. The threshold was set below the 0.3 μm bead cloud to exclude background noise. Samples were diluted 1:100 in sterile filtered PBS prior to analysis. Staining of platelet-derived EVs was performed with fluorescein isothiocyanate (FITC)-conjugated lactadherin (Haematologic Technologies Inc., Essex Junction, VT) as marker for EVs exposing phosphatidylserine in combination with a phycocyanin (PC7)-conjugated anti-CD41 monoclonal antibody as platelet marker (Beckman Coulter). To characterize EVs derived from other blood cells, we used phycoerythrin (PE)-conjugated anti-CD14 as monocyte marker, allophycocyanin (APC)-conjugated anti-CD235a as red blood cell marker, and pacific blue (PB)-conjugated anti-CD45 as leukocyte marker (all from Beckman Coulter). All antibodies were centrifuged at 17,000g for 5 min prior to staining to remove aggregates. Samples were measured for 3 min at a flow rate of 30 μl/min, and data were analyzed using the Kaluza Software (Beckman Coulter).

### Scanning electron microscopy

Following the circulation experiments, dialyzers were filled with 2.5% glutaraldehyde overnight, rinsed with isotonic saline, and opened with a saw. Individual fibers were removed and incubated in an ascending ethanol series (30% - 100%). Fibers were cut lengthwise with a scalpel under a microscope, fixed on a sample holder, sputtered with gold, and examined using a TM-1000 scanning electron microscope (Hitachi, Tokyo, Japan). A semiquantitative scoring system was employed to assess clot formation on the inner surface of the fibers as previously described [4]. Using a scale ranging from 0 to 4, (i) the area covered by the adhering cells or clots, (ii) formation of fibrin nets, (iii) presence of red blood cell aggregates, and (iv) adhesion of platelets were evaluated on 11–22 fibers per filter, and individual scores were added to obtain a total dialyzer clotting score.

### Statistical analysis

Data are presented as mean ± standard deviation (SD). Statistical analysis was performed using Statistica 7.0 (StatSoft Inc., Tulsa, USA). Biocompatibility parameters, measured at several time points, were compared between the groups using repeated measures analysis of variance (ANOVA); the main effects of time (as a repeated measure) and citrate group, as well as an interaction effect between them were analyzed. A Tukey HSD post-hoc test was used to compare time points to baseline values. Dialyzer clotting scores were compared between the groups using two-way ANOVA. EV counts were compared between the groups using Student´s T test. A p-value of < 0.05 was considered statistically significant.

## Results

### Citrate and calcium concentrations

Plasma concentrations of ionized calcium and citrate over the course of the experiment are given in Fig 1B and 1C. Baseline laboratory parameters for both citrate groups are summarized in Table 1. The initial decrease in citrate levels after the onset of circulation may be attributed to dilution by residual rinsing solution contained in the circuit as well as to diffusion of citrate across the dialyzer membrane. The measured plasma citrate concentrations (5.5 mM and 7.1 mM, respectively) differed from the target citrate concentrations in whole blood (3 mM and 4 mM), as citrate does not permeate red blood cell membranes and therefore is distributed in the plasma fraction only [23].

**Table 1:** Baseline laboratory parameters.

Parameter	3 mM citrate	4 mM citrate
hematocrit [%]	38.8 ± 2.6	39.3 ± 2.1
platelets [10^3^/μl]	192 ± 18	192 ± 25
ionized calcium [mM]	0.37 ± 0.03	0.27 ± 0.02
ionized magnesium [mM]	0.11 ± 0.01	0.08 ± 0.01
plasma citrate [mM]	5.52 ± 1.03	7.05 ± 0.68
mean platelet volume [fl]	9.6 ± 0.5	9.5 ± 0.6

### Influence of citrate concentration on biocompatibility parameters

**Fig 2:**
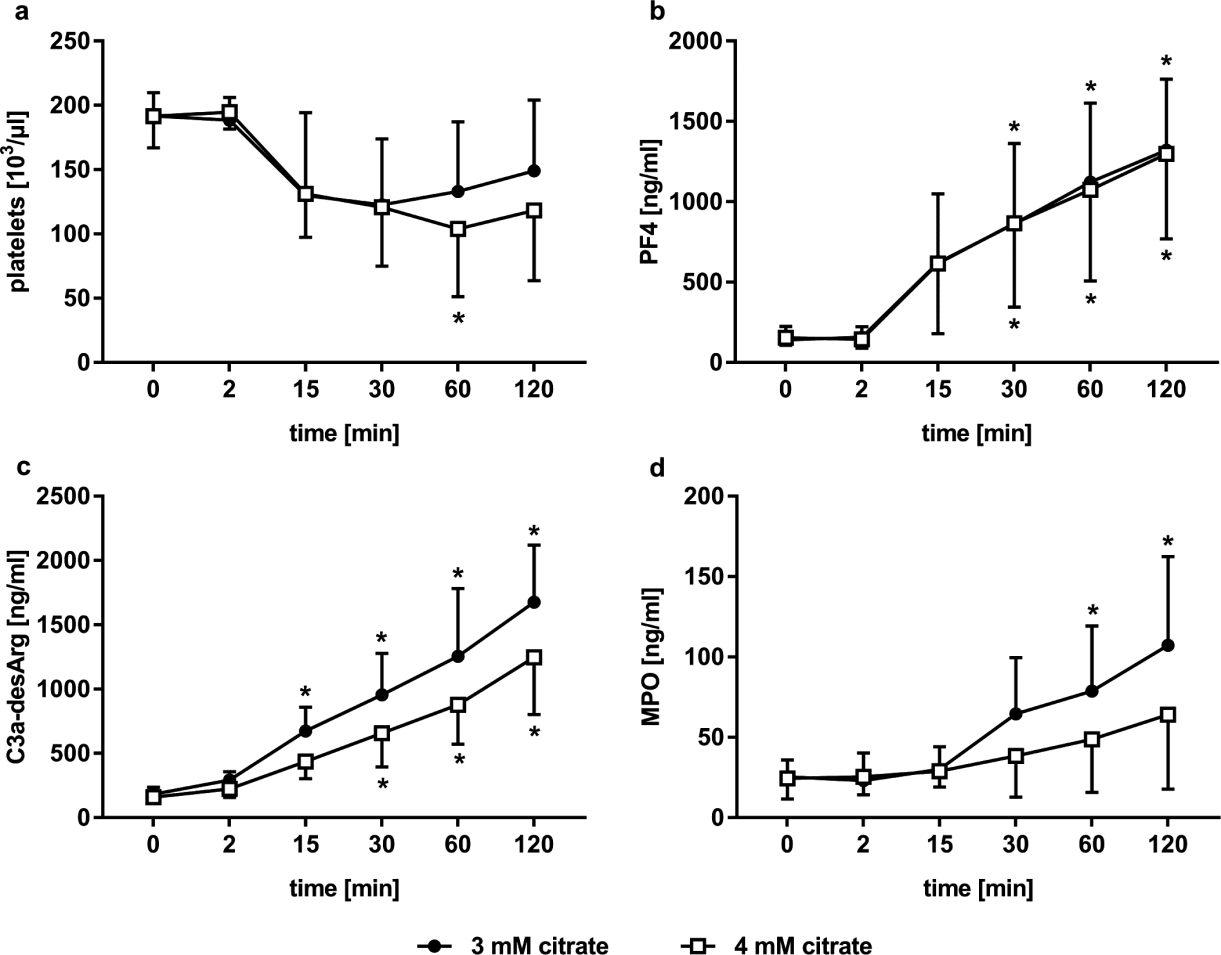
Activation of complement, platelets, and leukocytes during *in vitro* dialysis. (a) changes in platelet counts; (b) increase of soluble PF4 as marker of platelet activation; (c) release of C3a-desArg over time as marker of complement activation; (d) leukocyte activation, as indicated by MPO levels. Asterisks indicate significant (p < 0.05) changes of the respective parameters as compared to baseline levels; none of the parameters was significantly different between the groups.

Platelet counts decreased modestly within the first 15 min of recirculation and comparably for both citrate concentrations (main effect of time p < 0.001, citrate group p = 0.504 and interaction effect p = 0.679), indicating formation of platelet aggregates, and remained stable thereafter (Fig 2A). There was no effect of time on mean platelet volume (main effect of time p = 0.139, citrate group p = 0.525 and interaction effect p = 0.324; data not shown). Soluble PF4 increased steadily over the course of the experiment and comparably for both citrate concentrations (main effect of time p < 0.001, citrate group p = 0.950 and interaction effect p = 1.000). The increase as compared to baseline concentrations reached significance from 30 min onwards (Fig 2B). There was some increase in TBX2 levels over the course of the experiments, which was comparable in both groups (main effect of time p < 0.011, citrate group p = 0.968 and interaction effect p = 1.000; data not shown) and did not reach significance at any time point. C3a-desArg levels increased continuously from the onset of the experiment, indicating fast activation of the complement system. The increase reached significance from 15 min onwards in 3 mM group and from 30 min onwards in 4 mM group as compared to baseline levels, with a 9.2- and 7.8-fold increase after 120 min for 3 mM and 4 mM citrate, respectively (Fig 2C). C3a-desArg levels in the 4 mM citrate group tended to be lower as compared to the 3 mM citrate group, but the difference did not reach statistical significance (main effect of time p < 0.001, citrate group p = 0.107 and interaction effect p = 0.246). Levels of MPO, as a marker of leukocyte activation, increased continuously from 15 min onwards and were comparable between the groups (main effect of time p < 0.001, citrate group p = 0.234 and interaction effect p = 0.196). The increase as compared to baseline levels reached significance at 60 min for the 3 mM citrate group only (Fig 2D).

### Release of extracellular vesicles

**Fig 3:**
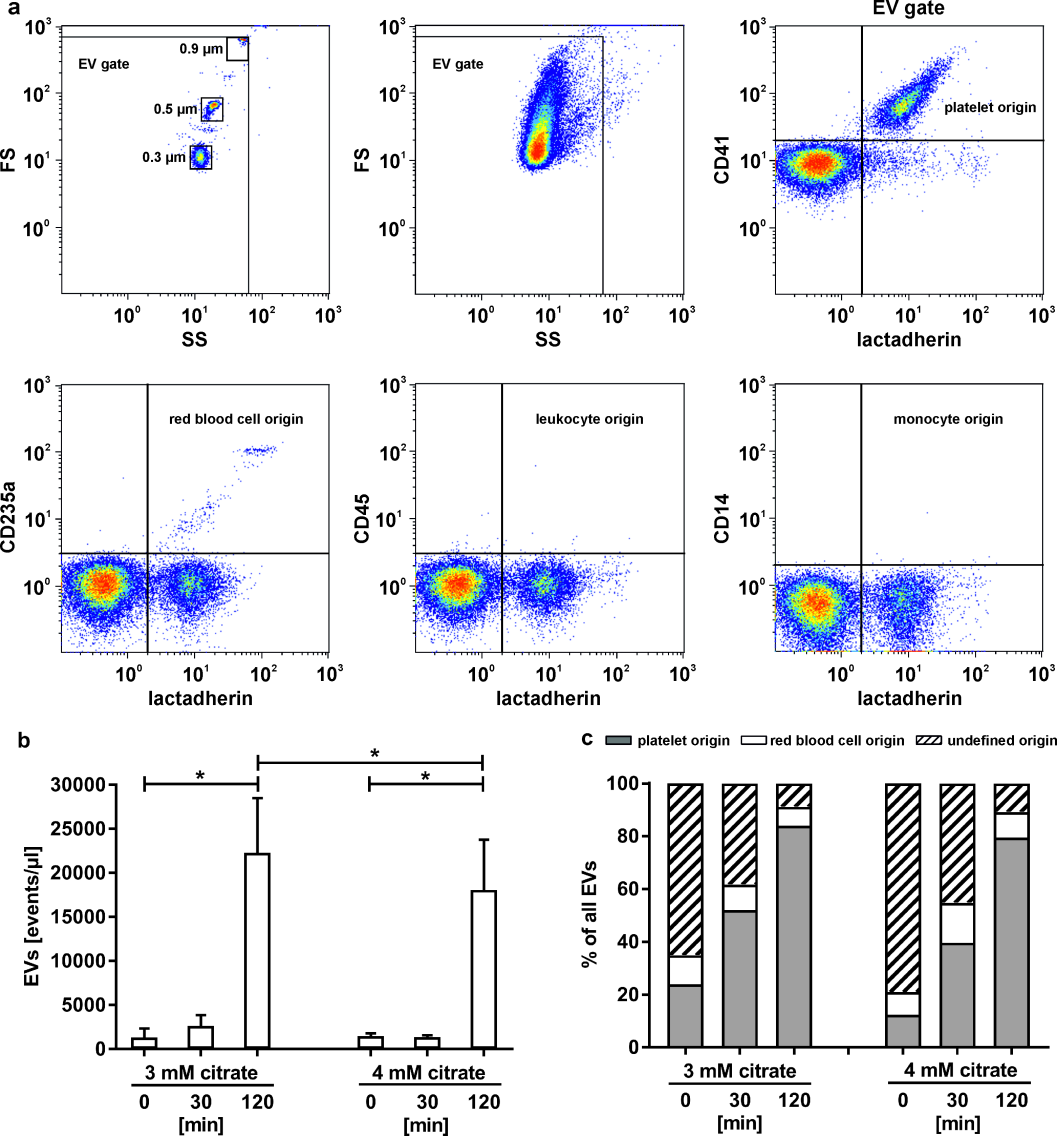
Release of extracellular vesicles. (a) Flow cytometric characterization and quantification of EVs was performed after calibration with fluorescent beads with diameters of 0.1, 0.3, 0.5, and 0.9 μm and the EV gate was set above the 0.9 μm bead cloud and the threshold was set below the 0.3 μm bead cloud to exclude background noise as described in the Methods section. EVs were identified by staining with lactadherin due to their exposure of phosphatidylserine. CD41, CD235a, CD45, and CD14 were used to trace platelet, red blood cell, or monocyte origin of EVs, as shown in representative lactadherin *vs*. cell surface marker dot plots; (b) increase in EV counts over time. The increase after 120 min was significant for both citrate groups as compared to baseline levels. The difference in EV counts between the two groups was significant at 120 min; (c) cellular origin of EVs in samples at the indicated time points, demonstrating that the large majority of EVs released during recirculation are platelet-derived (lactadherin^+^CD41^+^). EVs of undefined origin carry none of the cellular markers (CD41, CD235a, CD45, CD14). Statistically significant differences (p < 0.05) are marked by asterisks.

Flow cytometry was applied to quantify EVs as markers of cellular activation and to determine their cellular origin at 0, 30, and 120 min of circulation. Calibration was performed with fluorescent beads (0.1, 0.3, 0.5, and 0.9 μm), and EVs exposing phosphatidylserine were identified as lactadherin^+^ events in the EV gate, which was defined as shown in Fig 3A. EV counts increased significantly for both, 3 mM and 4 mM citrate, with a 17-fold increase in the 3 mM citrate group and a 12-fold increase in the 4 mM citrate group after 120 min, indicating the release of EVs during recirculation. The difference between the 3 mM and 4 mM citrate groups at 120 min was statistically significant (p = 0.022; Fig 3B). The large majority of EVs released during recirculation were CD41^+^, indicating their platelet origin, while EVs derived from red blood cells represented less than 10% of total EV counts at all time points (Fig 3C).

### Scanning electron microscopy

**Fig 4:**
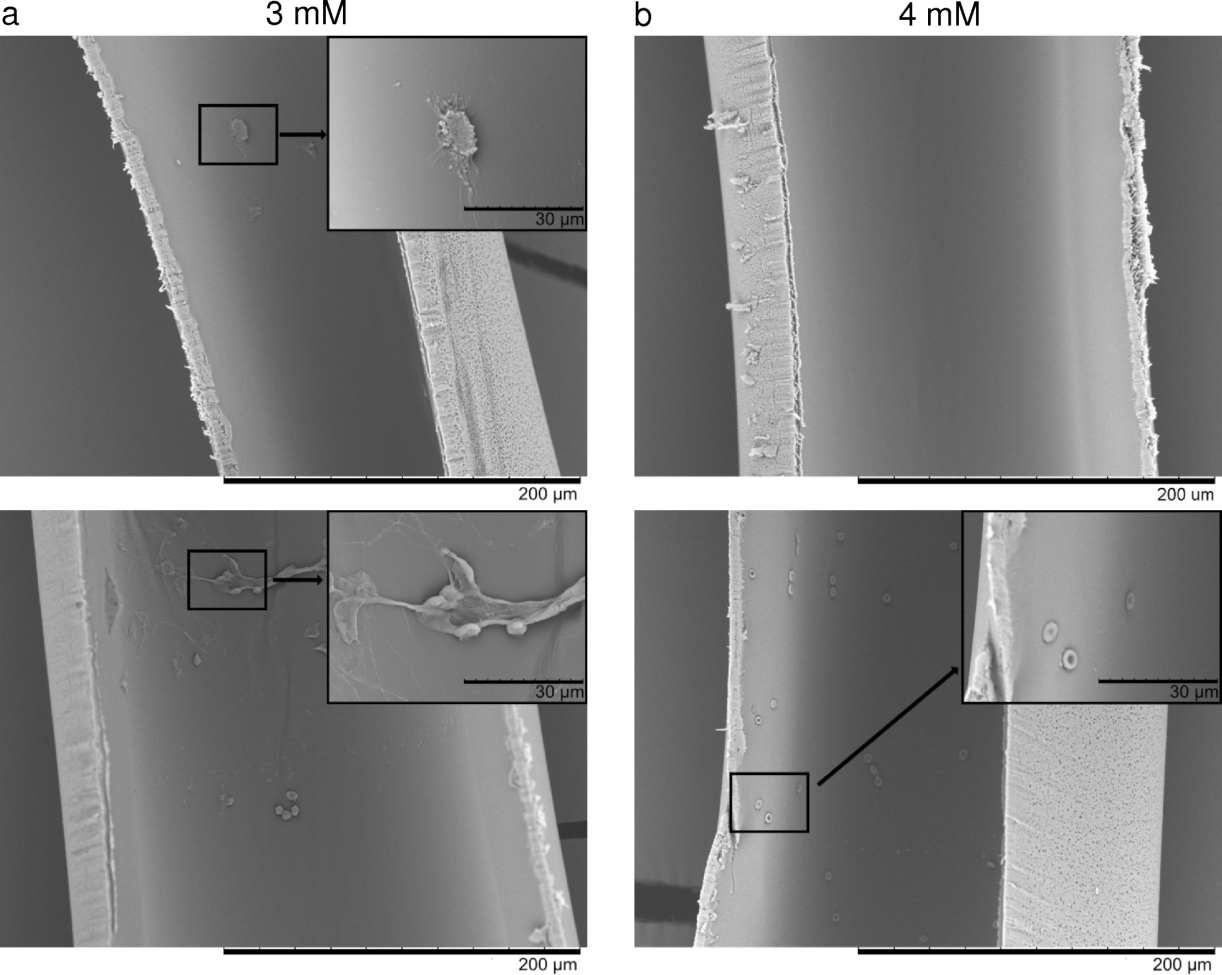
Representative scanning electron microscopy images of dialyzer fibers after *in vitro* dialysis in both citrate groups. (a) On fibers from the 3 mM citrate group, occasional platelets and small platelet aggregates (top) or red blood cells and fibrin deposition (bottom) was detectable. (b) Fibers from the 4 mM citrate group appeared almost entirely clean (top), with very low numbers of adhering red blood cells (bottom). Scale bars represent 200 μm (overview) and 30 μm (insert).

Adhesion of blood cells and activation of clotting was visualized using scanning electron microscopy of individual dialyzer fibers following the recirculation experiments. For semi-quantitative analysis, a dialyzer clotting score [4] was defined as described in the Methods section and summarized in Table 2. SEM revealed very low levels of cell adhesion for both citrate groups. Occasional platelet adhesion as well as formation of microclots was observed for the 3 mM citrate group, while only red blood cells where sporadically detectable on fibers from the 4 mM citrate group (Fig 4). The clotting score was significantly lower for the 4 mM citrate group as compared to the 3mM group (p = 0.046 for main effect of citrate group and p < 0.001 for main effect of experiment).

**Table 2:** Comparison of dialyzer clotting scores assessed by scanning electron microscopy.

Experiment	3 mM citrate	4 mM citrate
1	0.89 ± 1.59	0.20 ± 0.77
2	2.95 ± 3.01	1.91 ± 3.02
3	1.81 ± 4.71	0.23 ± 0.83
4	0.00 ± 0.00	0.00 ± 0.00
5	0.36 ± 0.50	0.00 ± 0.00
mean	1.34 ± 2.85	0.61 ± 1.83

## Discussion

In regional citrate anticoagulation, the depletion of ionized calcium in the extracorporeal circuit reduces many of the calcium-dependent blood-surface interactions, ensuring safe anticoagulation along with reduced complement and cellular activation. Here, we assessed the influence of citrate on a number of biocompatibility parameters in an *in vitro* dialysis set-up, focusing on a citrate concentration of 3 mM, which is commonly used in clinical protocols [13–15], and a higher concentration of 4 mM.

One of the first blood-surface interactions during hemodialysis is the activation of the complement cascade, which usually occurs within minutes after connection of the circuit, and, if severe, can cause clinically significant reactions [24]. While long-term effects related to complement activation during hemodialysis, such as systemic inflammation, have been greatly decreased with the introduction of synthetic dialysis membranes, the mode of anticoagulation has an impact on complement activation during dialysis, as well. Citrate has been reported to reduce activation of the complement system compared to heparin in apheresis [5], but data in dialysis remain contradictory [7, 25]. Only insignificant increases of complement have been observed during clinical dialysis with citrate concentrations of 3.4 mM [7]. In our *in vitro* study, complement activation, quantified by the release of C3a-desArg, occurred within minutes and was significant for both citrate concentrations, with a trend to lower activation in the 4 mM *vs*. the 3 mM citrate group. It is worth mentioning, however, that the experimental setting with a limited amount of blood used for prolonged recirculation, likely induced a higher extent of complement activation as compared to *in vivo* dialysis.

Similar to complement activation, platelet activation has been reported to occur in clinical dialysis with lower (2.4 mM [13]), but not with higher (3.4 mM [6]) doses of citrate. *In vitro*, we observed a significant increase of soluble PF4 for both the 3 mM and the 4 mM citrate groups, while the increase of TXB2 was much smaller, which might be attributed to the high transiency of TXB2, with a reported half-life of approximately 5 minutes [26]. The reduction in platelet counts observed in our study was likely caused by formation of platelet aggregates rather than by platelet deposition on the dialyzer, as evidenced by the absence of macroscopic clotting and by the fact that platelets were only scarcely detected on dialyzer fibers using scanning electron microscopy. As an additional marker of platelet activation, we quantified the release of platelet-derived extracellular vesicles, which have been associated with cellular activation in extracorporeal circuits [9, 21]. In addition to their pro-coagulant potential, which is mediated by their exposure of phosphatidylserine and, depending on the physiological setting, by the expression of tissue factor, EVs may exhibit pro-inflammatory effects, and their elevation has been confirmed in a number of pathological conditions [27–29]. We chose flow cytometric analysis for EV characterization despite its limited ability to detect EVs smaller than 300 nm, as it allows for the characterization of EVs with respect to their cellular origin based on surface marker expression. While platelet-derived EVs increased significantly during *in vitro* dialysis for both citrate groups, their levels remained significantly lower in the 4 mM *vs*. the 3 mM citrate group, providing further evidence for reduced cellular activation at higher citrate concentrations and confirming previous findings on reduced release of platelet-derived EVs at higher citrate concentrations in an *in vitro* set-up of lipoprotein apheresis[9].

Leukocyte degranulation during dialysis has been reported to occur independently of complement activation [7]. It has been shown to be reduced at citrate concentrations of 3.4 mM [7] in comparison to anticoagulation with heparin [7, 8], but there are no reports in the literature on leukocyte activation with protocols using lower doses of citrate. Our study showed a significant increase in MPO as compared to baseline levels in the presence of 3 mM citrate, indicating leukocyte activation over time. However, MPO levels did not significantly differ between the two citrate groups. Scanning electron microscopy to visualize microclots as well as blood cells adhering to the fibers of the dialyzers after the recirculation experiments revealed very low levels of cellular adhesion to the membranes for both citrate groups. For semi-quantitative analysis, we calculated clotting scores based on the presence and abundance of blood cells adhering to individual dialyzer fibers and to the presence of fibrin nets, as described in the Methods section, and were able to demonstrate that the total clotting score was significantly lower in the 4 mM citrate group as compared to the 3 mM group, although the mean clotting scores were excellent in both citrate groups compared to those reported with heparin and low-molecular weight heparins in the literature [4].

As a limitation of our study, recirculation of whole blood *in vitro* is not fully comparable to dialysis under *in vivo* conditions. In particular, dialysis under our experimental conditions required the addition of heparin to avoid clotting of the extracorporeal circuit, which at least partly precludes the full attribution of the results to the effect of citrate, regardless of the low dose of heparin that was added to supplement citrate anticoagulation.

To conclude, our results do point to a further improvement in biocompatibility with increasing citrate dose from 3 mM to 4 mM, and one could argue that a further increase of the citrate concentration might completely suppress cellular activation in the extracorporeal circuit. Its clinical use, however, would be limited, taking into account the risk of citrate accumulation, particularly with high blood flow, such as in intermittent hemodialysis. A concentration of 4 mM citrate might therefore be optimal in clinical practice, but this remains to be confirmed in the clinical setting.

## Supporting information

S1 File Raw study data. (XLS)
